# Supplementary material for: Metabolic Biomarkers Assessed with PET/CT Predict Sex-Specific Longitudinal Outcomes in Patients with Diffuse Large B-Cell Lymphoma
Source: Cancers (Basel). 2022 Jun 14;14(12):2932. doi: 10.3390/cancers14122932 (PMC9221486; doi:10.3390/cancers14122932)
Supplement: Supplementary file 1 [file cancers-14-02932-s001.zip › cancers-1691028-supplementary.pdf]

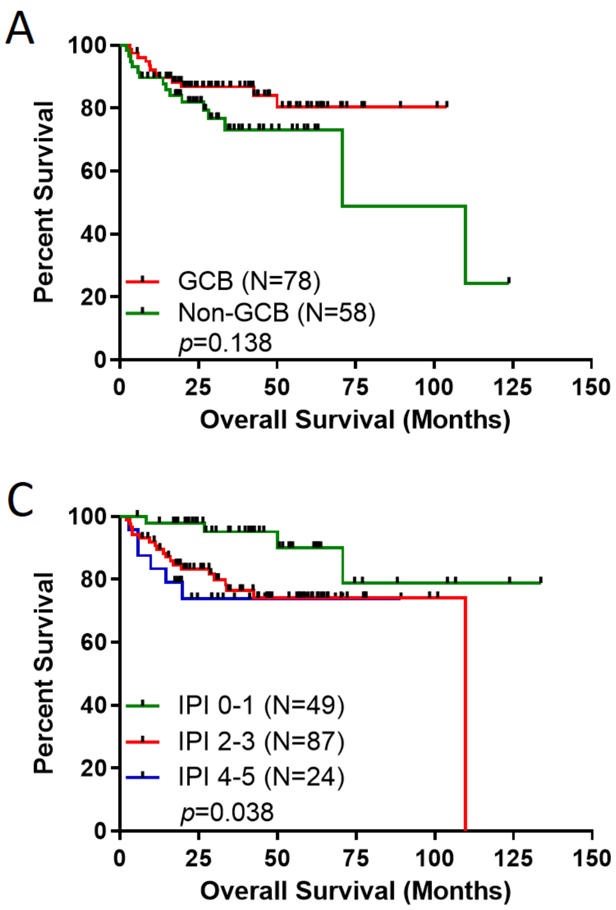

Figure S1. Impact of DLBCL prognostic classifiers on patient survival in this cohort. A. Overall survival and B. Progression free survival for germinal center B-cell like (GCB) and non-GCB subtypes. C. Overall survival and D. Progression free survival for International Prognostic Index (IPI) scoring. *p*-value was calculated with the log-rank test.

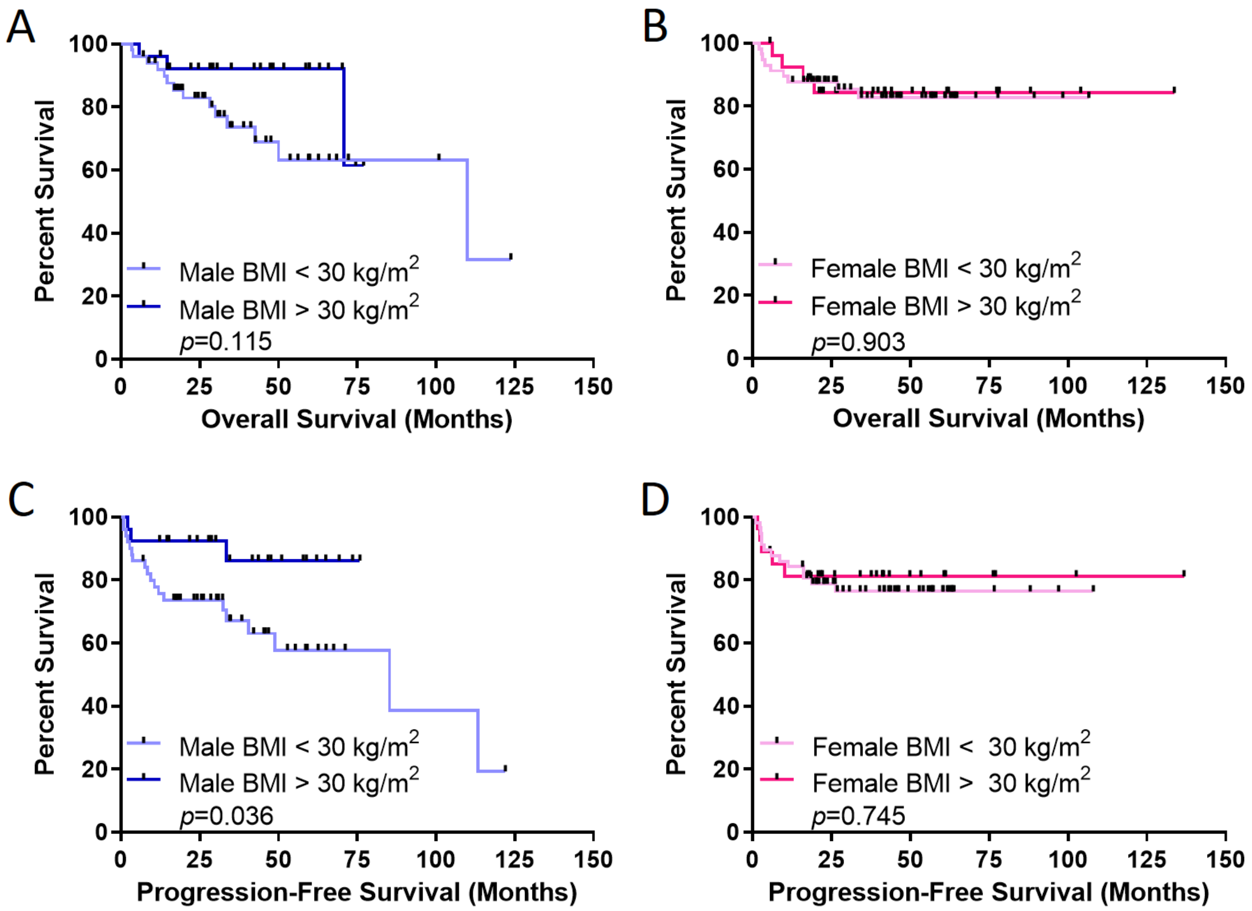

Figure S2. Impact of obese BMI on patient survival in this cohort. Overall survival (OS) in A. males and B. females and Progression free survival in C. males and D. females with obese versus non-obese BMI (i.e., 30 kg/m^2 threshold). *p*-value was calculated with the log-rank test.

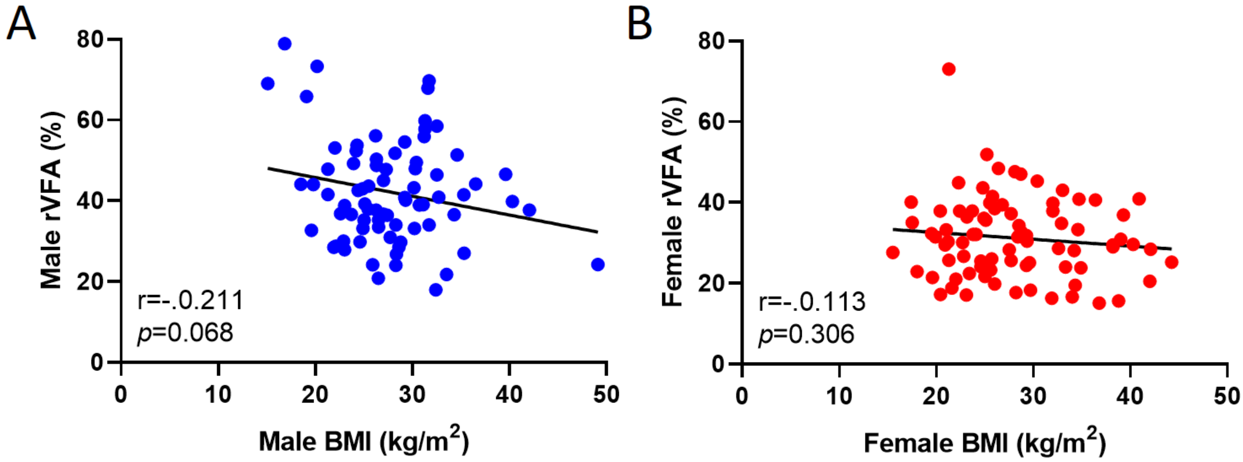

Figure S3. Correlation analyses of BMI and rVFA in this cohort. No significant correlations exist between these variables in either A. males or B. females. r = Pearson correlation coefficient. .

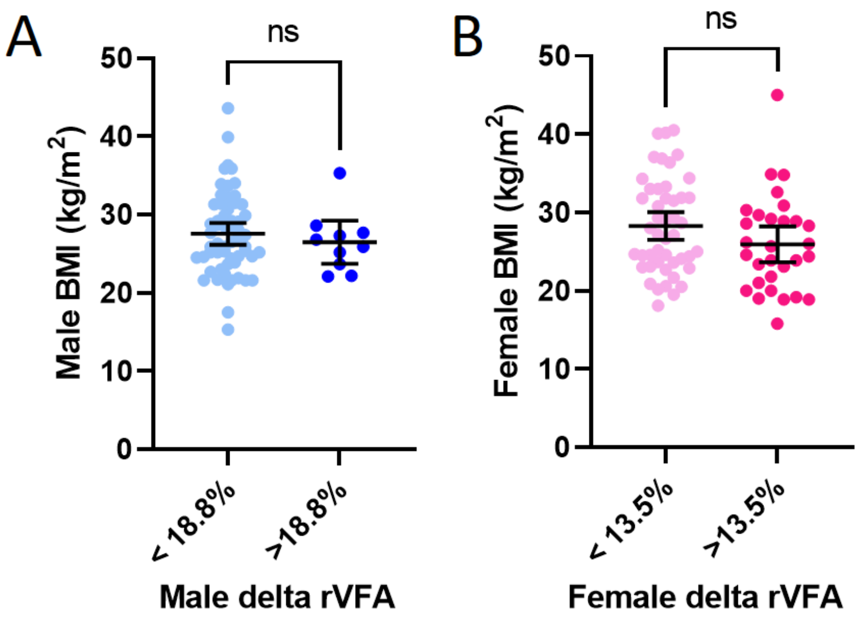

Figure S4. Effect of delta rVFA groups on BMI in this cohort. A. Males, B. Females. Delta rVFA groups were derived from the optimized delta rVFA threshold in Figure 3. *p*-value calculated with 2-tailed Mann Whitney test. ns = not significant.

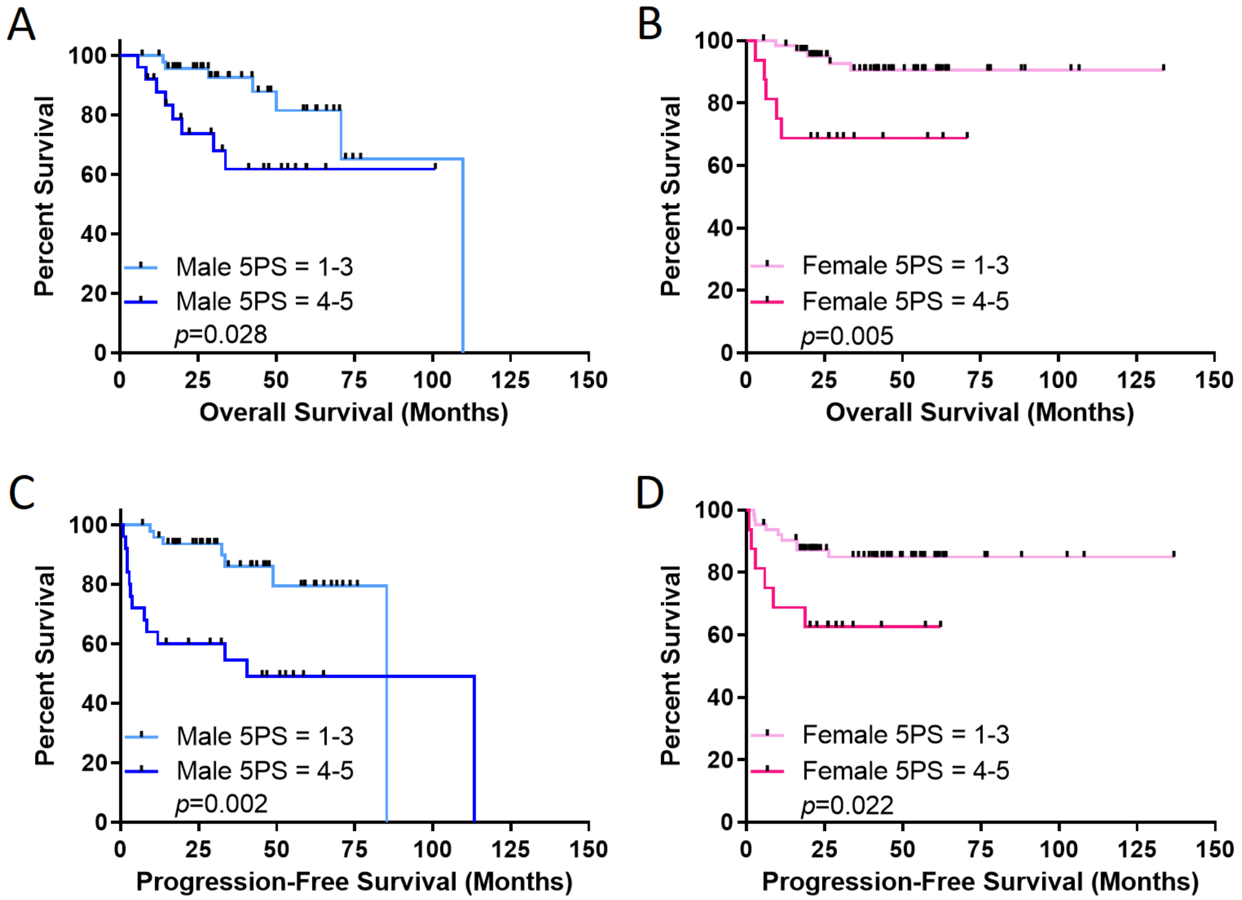

Figure S5. High 5PS score is associated with decreased OS and PFS in males and females. 5PS score at end of treatment was dichotomized into low (1-3) and high (4-5) groups. *p*-value was calculated with the log-rank test. .

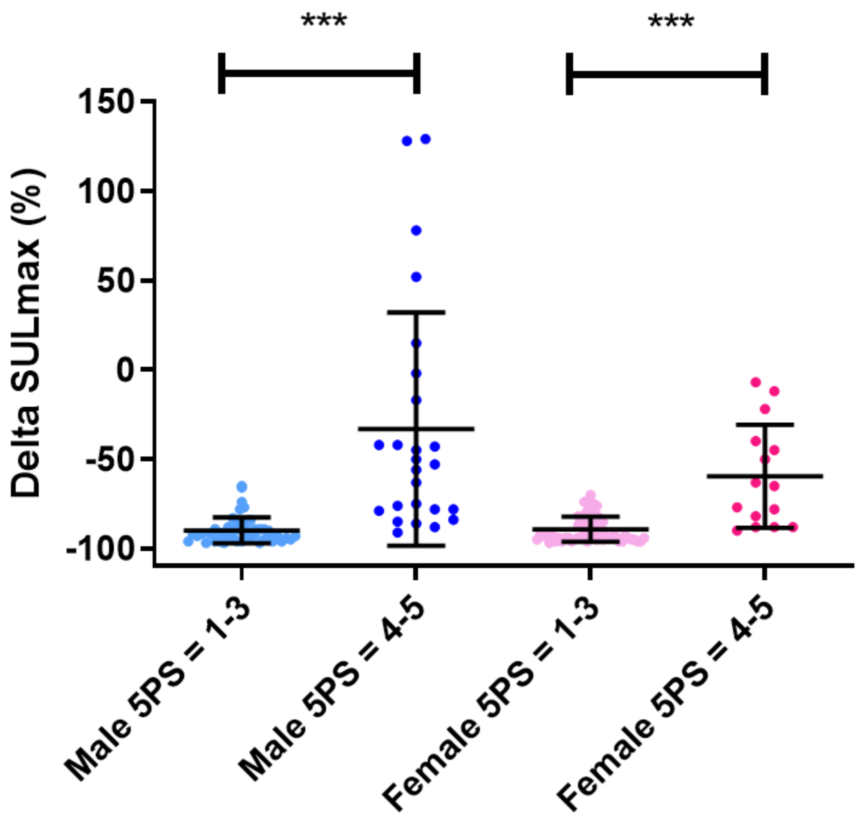

Figure S6. Higher 5PS scores at end of treatment are associated with increased delta SULmax values. *p*-value calculated with 2-tailed Mann Whitney test. ***, $p < 0.001$.

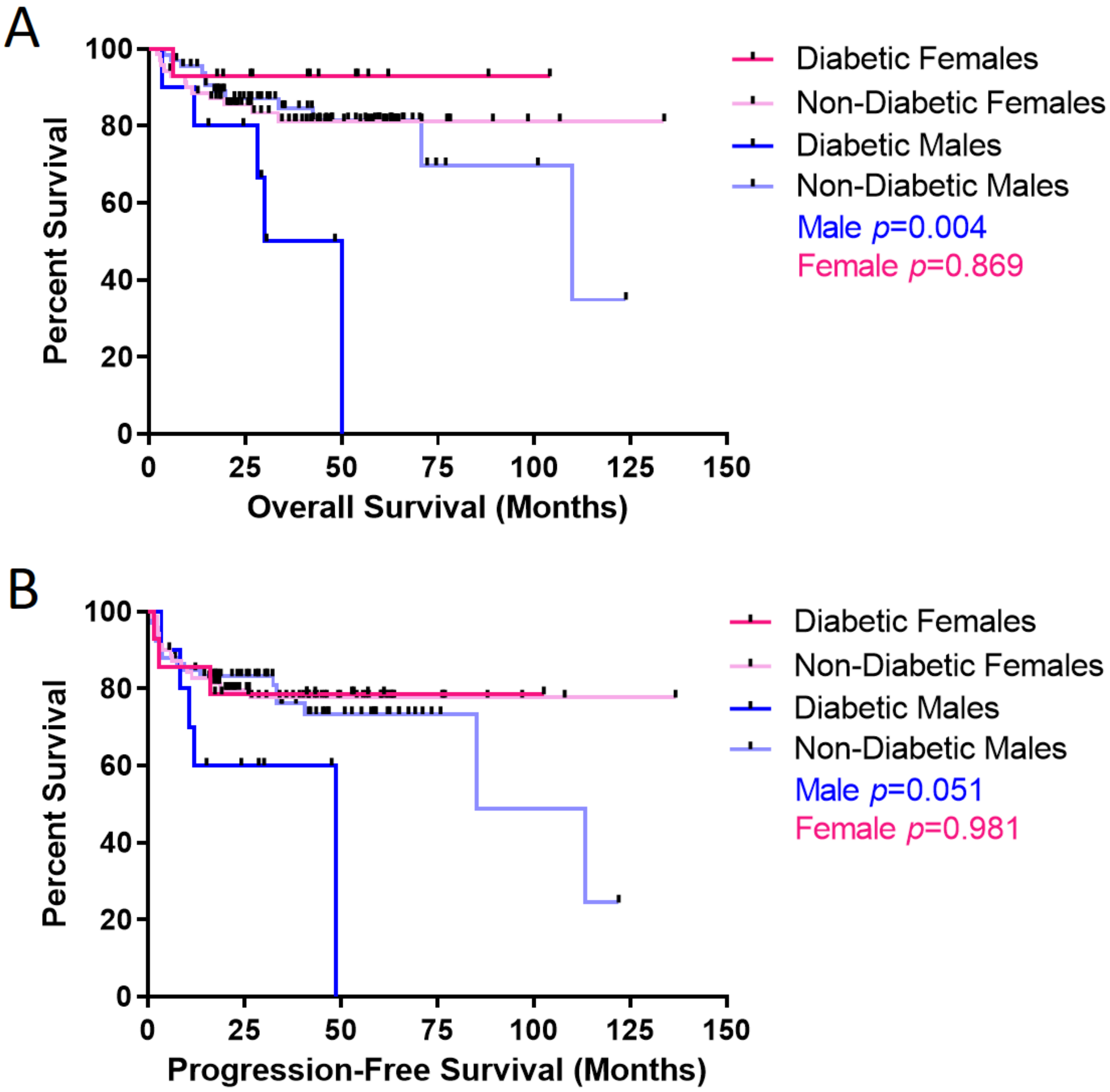

Figure S7. In DLBCL, males with diabetes have significantly worse survival compared to females. A. Overall survival and B. Progression free survival. *p*-value was calculated with the log-rank test. .
